# Generate what you can make: achieving in-house synthesizability with readily available resources in de novo drug design

**DOI:** 10.1186/s13321-024-00910-4

**Published:** 2025-03-28

**Authors:** Alan Kai Hassen, Martin Šícho, Yorick J. van Aalst, Mirjam C. W. Huizenga, Darcy N. R. Reynolds, Sohvi Luukkonen, Andrius Bernatavicius, Djork-Arné Clevert, Antonius P. A. Janssen, Gerard J. P. van Westen, Mike Preuss

**Affiliations:** 1 Leiden Institute of Advanced Computer Science, Leiden University, Leiden, The Netherlands; 2 Leiden Academic Centre of Drug Research, Leiden University, Leiden, The Netherlands; 3 CZ-OPENSCREEN: National Infrastructure for Chemical Biology, Department of Informatics and Chemistry, Faculty of Chemical Technolog, University of Chemistry and Technology Prague, Prague, Czech Republic; 4 Leiden Institute of Chemistry, Leiden University, Leiden, The Netherlands; 5 Machine Learning Research, Pfizer Research and Development, Berlin, Germany

**Keywords:** Computer-aided synthesis planning, Casp, Retrosynthesis, Synthesizability, Synthesizability score, De novo drug design, Virtual screening, In vitro, Medicinal chemistry

## Abstract

Computer-Aided Synthesis Planning (CASP) and CASP-based approximated synthesizability scores have rarely been used as generation objectives in Computer-Aided Drug Design despite facilitating the in-silico generation of synthesizable molecules. However, these synthesizability approaches are disconnected from the reality of small laboratory drug design, where building block resources are limited, thus making the notion of in-house synthesizability with already available resources highly desirable. In this work, we show a successful in-house *de novo* drug design workflow generating active and in-house synthesizable ligands of monoglyceride lipase (MGLL). First, we demonstrate the successful transfer of CASP from 17.4 million commercial building blocks to a small laboratory setting of roughly 6000 building blocks with only a decrease of –12% in CASP success when accepting two reaction-steps longer synthesis routes on average. Next, we present a rapidly retrainable in-house synthesizability score, successfully capturing our in-house synthesizability without relying on external building block resources. We show that including our in-house synthesizability score in a multi-objective *de novo* drug design workflow, alongside a simple QSAR model, provides thousands of potentially active and easily in-house synthesizable molecules. Finally, we experimentally evaluate the synthesis and biochemical activity of three *de novo* candidates using their CASP-suggested synthesis routes employing only in-house building blocks. We find one candidate with evident activity, suggesting potential new ligand ideas for MGLL inhibitors while showcasing the usefulness of our in-house synthesizability score for *de novo* drug design.

**Scientific contribution** Our core scientific contribution is the introduction of in-house *de novo* drug design, which enables the practical application of generative methods in small laboratories by utilizing a limited stock of available building blocks. Our fast-to-adapt workflow for in-house synthesizability scoring requires minimal computational retraining costs while supporting a high diversity of generated structures. We highlight the practicality of our approach through a comprehensive in-vitro case study that relies entirely on in-house resources, including in-silico generation, synthesis planning, and activity evaluation.

**Supplementary Information:**

The online version contains supplementary material available at 10.1186/s13321-024-00910-4.

## Introduction

In drug discovery, the traditional Design-Make-Test-Analyze (DMTA) cycle is undergoing substantial changes, driven by the incorporation of novel artificial intelligence approaches [1]. Within the “Design” phase of DMTA, *de novo* drug design methods have emerged that propose novel molecular structures, already demonstrating effectiveness in identifying potential new drug candidates [2, 3]. In this search process, optimization-based *de novo* methods repeatedly generate a selection of candidate molecules, evaluate these candidate molecules with desired objective functions, and optimize the generative method towards desired chemical spaces [4, 5]. Inherently, this search involves multi-objective optimization, as generated molecules should satisfy various potentially contradicting and, therefore, non-combinable objectives (i.e., selectivity for the desired protein target, pharmacokinetic properties, or synthetic accessibility) [6].

Simultaneously, the conceptualization of the “Make” phase of DMTA has also undergone massive changes with the emergence of artificial intelligence approaches, where Computer-Aided Synthesis Planning (CASP) determines synthesis routes by deconstructing molecules recursively into molecular precursors until a collection of commercially available molecules, commonly termed “building blocks”, is identified [7, 8]. Rather than manually searching for these synthesis routes, contemporary approaches employ neural networks to encapsulate the backward reaction logic and search algorithms to find possible multi-step reaction pathways [9].

One of the existing challenges limiting the broader adoption of *de novo* techniques in the “Design” phase is the generation of unrealistic, non-synthesizable molecular structures. Here, different strategies have become available to include synthesizability to ensure realistic molecular structures [10]. The most straightforward approach is to directly use synthesis planning, assessing if a synthesis route can be found using one of the available approaches [7, 11–13]. Lately, this approach has been successfully investigated as an objective in *de novo* drug design [14], but has high computational requirements and is time-intensive [4, 10]. In this scenario, each molecule necessitates an entire synthesis planning run, where the duration can range from minutes to several hours depending on the selected retrosynthesis neural network [15, 16]. Unfortunately, this renders synthesis planning incompatible with most optimization-based *de novo* drug design methods, as these methods require numerous optimization iterations to achieve convergence.

A more efficient alternative to full synthesis planning is the use of synthesizability heuristics or learned synthesizability scores that (indirectly) provide a fast measure of synthesizability, making them well suited for post-generation virtual screening or *de novo* drug design [4, 10].

These synthesizability heuristics can be as simple as the length of the SMILES string [10], the presence of fragments typical in synthesizable molecules [17], or the combination of typical structural features of synthesizable molecules with a penalty for structural complexity like rings or stereo-centers [18]. Within *de novo* drug design, these heuristics are occasionally used as generation objectives to improve synthesizability (e.g., [10]) or as post-generation filters to identify synthetic accessible molecules (e.g., [17, 19]).

In contrast to synthesizability heuristics, CASP-based synthesizability scores approximate synthesis planning results and learn the relationship between a molecule’s structure and the successful identification of a synthesis route via synthesis planning [20]. This learning task is either formulated as a classification task of the synthesis planning outcomes [20, 21] or a regression task relying on the resulting synthesis route properties [14, 22]. However, these CASP-based scores are thus far rarely used as an objective in *de novo* drug design and are missing in common *de novo* benchmark frameworks (e.g., [23]).

Nevertheless, the limited in-silico studies that use the aforementioned CASP-based scores indicate: First, they improve synthesizability in terms of the used score in an in-silico *de novo* drug design benchmark [22] but lack in-silico evaluation of potential synthesis routes. Second, they improve post-generation synthesis planning success in an in-silico lead optimization benchmark [14] but lack the experimental evaluation of generated structures and synthesis routes.

All of the above ties into a common challenge of the field, where contemporary *de novo* drug design and synthesizability approaches do not take the experimental reality of drug discovery into account, as most *de novo* approaches are evaluated against synthesizability and activity heuristics (e.g., [23]) instead of synthesizing potential drug candidates and measuring their activity experimentally [24]. This absence of experimental evaluation and focus on computational benchmarking environments is also present in *de novo* methods that explicitly include synthesizability scores to actively enforce realistic and synthetically accessible molecular structures (e.g., [14, 22]), yielding the question of whether suggested approaches also work experimentally regarding the proposed drug candidates and the suggested synthesis routes.

In addition to the lack of experimental evaluation, these general CASP-based synthesizability scores assume near-infinite building block availability. This assumption is, however, far removed from a realistic laboratory setting, where resources are limited regarding budget and lead times for building blocks, making a specific notion of in-house synthesizability tailored to available resources more valuable than a general notion of synthesizability. However, this transfer of contemporary CASP methods, which rely on millions of commercially available building blocks, to a resource-limited environment might be challenging for two reasons: First, the CASP performance is limited by the quantity and nature of available building blocks, where missing building blocks can lead to unsolvable molecules [20]. Second, current CASP-based synthesizability scores are not building block agnostic as they create their training data to capture a general notion of synthesizability with these millions of commercially available building blocks (e.g., [14, 20, 22]).

In this work, we address both of those challenges in the field of computer-aided *de novo* drug design:

- First, we demonstrate the successful transfer of synthesis planning to an environment with a limited in-house collection of building blocks, revealing that an extensive commercial inventory is unnecessary for identifying potential synthesis routes. Specifically, we show that using only 6,000 in-house building blocks results in merely -12% loss in synthesis planning performance for a large drug-like chemical space, compared to employing a roughly 3000-fold more extensive library of commercially available building blocks (“Zinc” [11]).
- Second, we introduce an in-house CASP-based synthesizability score that can successfully predict if molecules are synthesizable with our in-house building blocks. We establish that a well-chosen dataset of 10,000 molecules suffices for training this score, allowing rapid retraining to accommodate changes in building blocks through iterative synthesis planning and model training.
- Third, we demonstrate the effectiveness and usefulness of both in-house and general CASP-based synthesizability scores within *de novo* drug design. When combined with a MGLL [25] protein target QSAR model as objectives, we show that the in-house synthesizability score facilitates the generation of thousands of in-house, easy-to-synthesize and potentially active drug candidate molecules.
- Finally, we experimentally evaluate and critically analyze three generated molecules using an in-house synthesizability score after synthesis based on AI-suggested, in-house CASP routes. In the process, we find one active candidate, suggest potential novel ligand ideas for MGLL inhibitors, and examine differences between our experimentally evaluated molecules, the generated in-house candidate space, and known MGLL ligands.

## Results and discussion

### In-house synthesizability

**Fig. 1:**
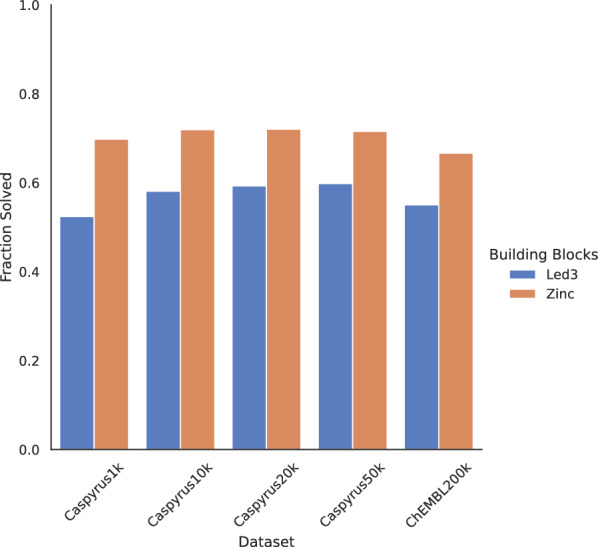
Synthesis Planning Performance. Evaluation using 5955 Leiden University in-house (“Led3”) or 17.4 million general building blocks (“Zinc”). Percentage of molecules where a complete synthesis route to either building blocks can be found using synthesis planning on different subsets of a Butina-clustered Papyrus [26] (“Caspyrus”) or a sample of 200,000 ChEMBL molecules

To evaluate the transfer synthesis planning to our real-life, resource-limited university setting, we deployed the open-source synthesis planning toolkit AiZynthFinder [11, 37] with two different building block sets, 5,955 in-house university building blocks (“Led3”) and 17.4 million generally available commercial compounds (“Zinc”). The synthesis planning performance was evaluated for two datasets, a set number of centroids of a Butina-clustered [27] subset from Papyrus (“Caspyrus”) [26] and a set of 200,000 randomly sampled drug-like ChEMBL [28] molecules.

**Fig. 2:**
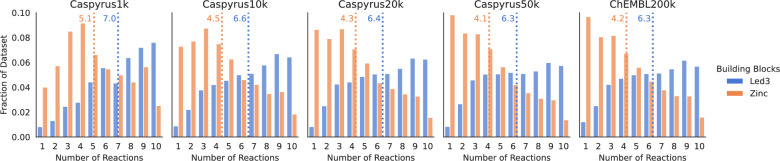
Distribution of the shortest synthesis route found. Evaluation using synthesis planning with 5,955 building blocks (Led3) and 17.4 million building blocks (Zinc) on the Caspyrus and 200,000 ChEMBL molecules datasets. The dotted line indicates the average route length for both building block sets

An overview of the synthesis planning results is presented in Fig. 1. This analysis showed that the difference in performance when using only 5955 Led3 building blocks compared to 17.4 million Zinc building blocks, despite a 3000-fold increase, is notably small. Using the more limited Led3 building blocks, solvability rates for Caspyrus centroids are around 60%, except when using only 1000 clusters (“Caspyrus1k”) or evaluating on ChEMBL. For the far more extensive Zinc building blocks, solvability rates are around 70% across all datasets. The solvability disparity between both building blocks is around +12% for most datasets except for Caspyrus1k, where roughly +17% more molecules are solved with Zinc building blocks. A notable difference between both building blocks is that the shortest synthesis route found with in-house building blocks is, on average, two reaction steps longer than those using Zinc building blocks, as more building blocks allow shorter synthesis routes across all datasets (see Fig. 2). Surprisingly, the increase in synthesis route length is relatively uniform across all molecules for both the Caspyrus50k and Chembl200k datasets, as no distinct areas of the chemical space require longer synthesis routes or are unsolvable when using in-house building blocks (see Supplementary: Fig. A1, A2).

Overall, these results suggest that storing a large commercially sized stock of building blocks is unnecessary to run synthesis planning, as a small building set loses only – 12% solvability when accepting slightly longer synthesis routes. These results open the possibility of planning the synthesis of desired compounds in-house instead of buying new building blocks from a vendor and potentially allowing the prioritization of interesting drug discovery candidates according to available in-house resources.

### In-house synthesizability score

**Fig. 3:**
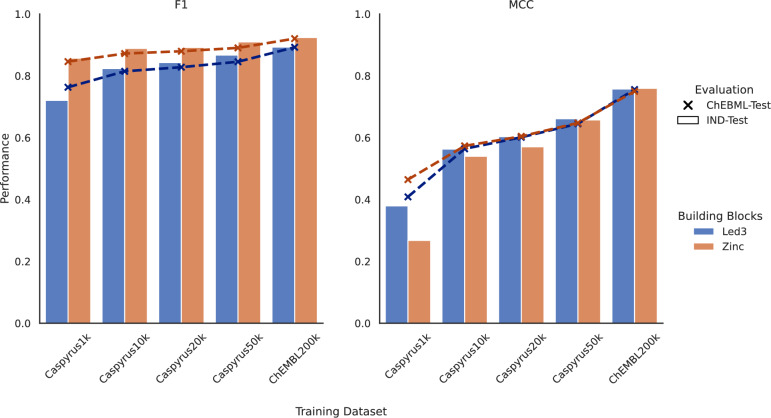
Benchmarking in-house and general synthesizability scores. Performance comparison of CASP-based synthesizability scores predicting the synthesizability using in-house (“Led3”) and general (“Zinc”) building blocks in contrast to finding a synthesis route using synthesis planning. Scores are evaluated by measuring the F1 and MCC scores on independent test sets of the respective training datasets (“IND-TEST”) and 200,000 newly sampled and, to all models, unknown ChEMBL molecules (“ChEMBL-TEST”)

After discovering that in-house building blocks are sufficient for performing synthesis planning, we trained a CASP-based synthesizability score for assessing the in-house synthesizability of molecules without requiring resource-intensive synthesis planning. In short, we trained an XGBoost model [29], following the methodology suggested by RaScore [20], to predict if a complete synthesis route can be found for a molecule using synthesis planning. Here, we used the previously generated routes for the in-house Led3 and Zinc building blocks as training data. Afterward, we evaluated the models on respective independent test sets (10% of the data - “IND-Test”) and 200,000 newly sampled ChEMBL molecules not present in any training datasets (“ChEMBL-Test”) to further evaluate generalizability, for which we additionally conducted synthesis planning with both building block sets (Fig. 3).

On both evaluation tasks, our trained in-house models achieved excellent results in both F1 and Matthews Correlation Coefficient (MCC) [30, 31] classification scores, which were used to assess the predictivity of synthetic accessibility by the trained scorer. For datasets with at least 10,000 molecules, the F1 performance on the respective test sets surpassed 0.8, proving competitive with the results from larger training datasets. The MCC performance generally improved with more training data, reaching acceptable levels with at least 10,000 molecules, likely because more data enhances the discernment of non-synthesizable molecules. When employing the same training data but using routes based on Zinc building blocks instead, the resulting classifiers performed comparably to those trained with in-house building blocks. Like the Led3 building blocks, classifiers based on Zinc building blocks achieved acceptable F1 and MCC performance when trained on datasets of at least 10,000 molecules. The performance differences in F1 and MCC between the respective dataset test sets and the additionally sampled and unseen 200,000 ChEMBL molecules were minor (except for Caspyrus1k).

These results indicate that our models can accurately estimate in-house synthesizability on a large drug-like chemical space and generalize beyond their respective test sets, allowing us to assess in-house synthesizability for our laboratory in the drug discovery process.

### In-house synthesizability of generated molecules

Since we can successfully predict if a molecule is in-house synthesizable, we wanted to investigate if these scores can be used in a *de novo* drug design setting to generate in-house synthesizable drug candidates.

For this purpose, we combined our in-house synthesizability scores with an MGLL QSAR model to train a multi-objective DrugEx [19] molecular generator to find potent and readily synthesizable compounds for this target (compare training details in methods "De novo molecular generation" section). We deployed a novel DrugEx training strategy that helped our generator to learn the desired chemical spaces by guiding it from a general drug-like chemical space towards our target space with both a fine-tuned target-specific generator model, capturing the known ligand distribution, and a QSAR model, capturing the scaffold specific information. As we wanted to evaluate the effect of different synthesizability scores, we trained multiple molecular generators with different QSAR and synthesizability model combinations. We used the QSAR model without any synthesizability score or in combination with either the SAScore [18] or our in-house and general synthesizability scores trained on 10,000 and 200,000 molecules (Caspyrus10k & ChEMBL200k). To evaluate the trained molecular generators, we sampled 100,000 molecules for each trained generator and assessed how many are synthesizable with either building blocks using synthesis planning (“solved”) and are seen as active by the QSAR model with a probability larger than 0.8 (“active”).

**Fig. 4:**
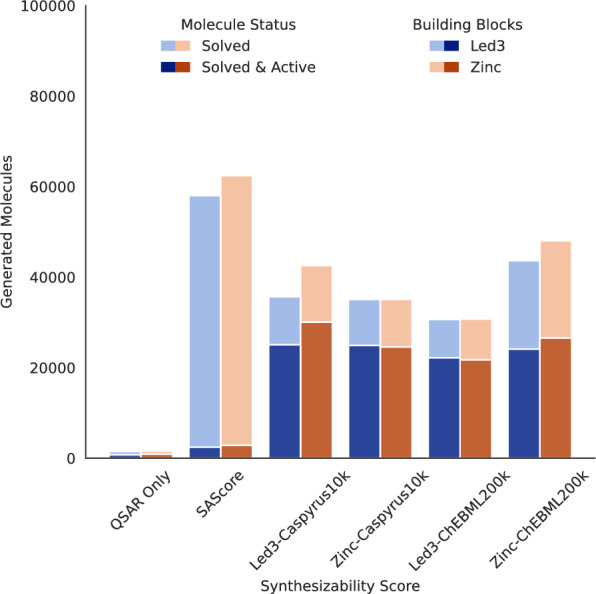
Generated synthesizable and potentially active molecules using in-house synthesizability scores. Evaluation of 100,000 molecules generated per selected QSAR model and CASP-based synthesizability score combination. “Solved” denotes the successful identification of a synthesis route for a particular molecule with the respective building blocks (in-house Led3 and Zinc), while “Active” is measured by the QSAR model with a probability threshold of greater than 0.8

The performance of different synthesizability scores in combination with our QSAR model is presented in Fig. 4. Compounds generated with only a QSAR objective have a very low yield of solvable and active structures since the generative model is not guided by synthesizability constraints. Here, generated structures tend to exploit the QSAR model (i.e., repeat the active structural patterns to increase the probability of being flagged as active) but are synthetically inaccessible when solving with both the in-house and general building blocks. In contrast, adding SAScore as an objective produces many solvable but very few active molecules, as most generated structures are too structurally constrained by SAScore to be active but are consequently easy to synthesize. Regarding synthesizability scores trained using synthesis planning, all CASP-based synthesizability scores perform well and produce between 20,000 and 30,000 predicted active and synthesizable candidates using either the in-house or general building blocks. Surprisingly, scores trained on Caspyrus10k produce the most solved and active molecules, whereas CASP-based synthesizability scores trained on 200,000 ChEMBL molecules produce more solved molecules but not more active ones. It is worth noting that the solvability of the generated molecules is expectably lower than the ChEMBL test sets (compare Fig. 3) as molecules are generated along the Pareto front between the QSAR model and the respective used synthesizability score (compare Supplementary: Fig. C9 for an example of the generated objective space).

Quantitatively, our experiment shows that using in-house synthesizability scores within a *de novo* generator can produce thousands of in-house synthesizable molecules, which can function as a starting point for experimental in-house evaluation.

### Synthesizability score impact on generated molecules

After we showed that CASP-based synthesizability scores facilitate the generation of synthesizable molecules, we set out to investigate their impact on the generated candidates and potential problems with their predictive performance in the desired candidate space.

**Fig. 5:**
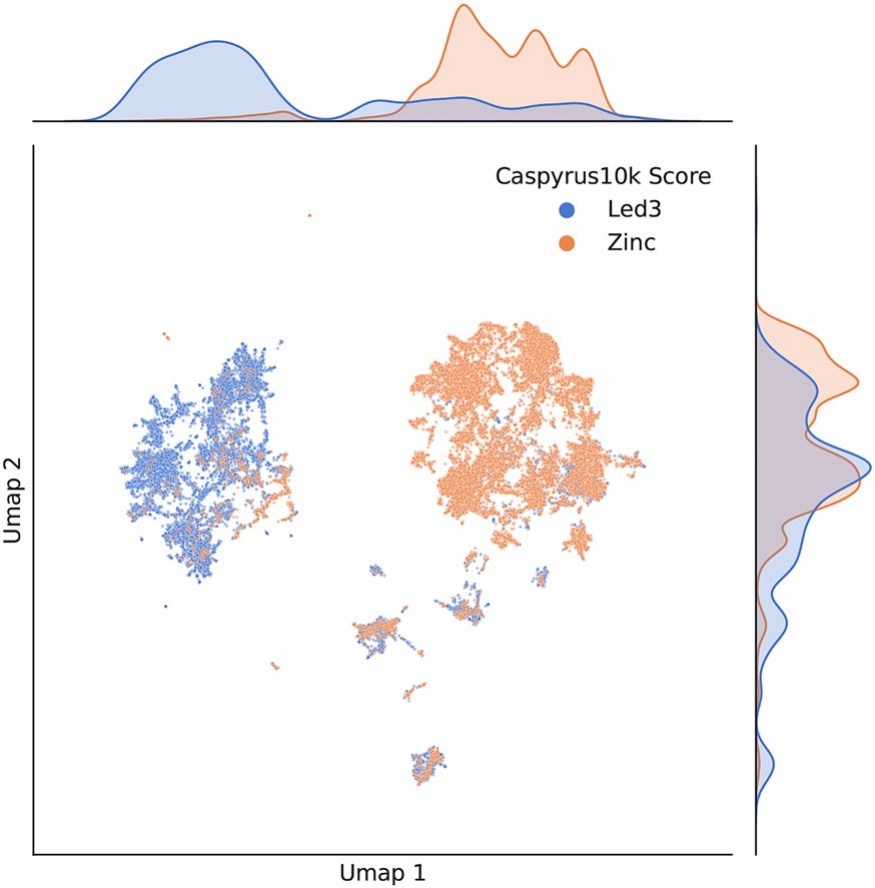
Contrasting the shared generated chemical space of in-house and general synthesizability scores. UMAP visualization of the solved and potentially active molecular space derived from combining the molecules generated from both in-house and general synthesizability scores trained on the same dataset (“Caspyrus10k”). In both instances, in-house building blocks are used for synthesis planning to evaluate solvability. UMAP is calculated using Morgan Fingerprints (Radius 3, Size 2048)

First, given that we tested in-house and general synthesizability scores alongside our QSAR model, an obvious question is whether these different scores target separate chemical spaces and generate, consequently, distinct candidates. Our primary motivation stems from the fact that the number of solved *de novo* candidate molecules from the in-house and general Caspyrus10k synthesizability scores are comparable when using in-house building blocks within synthesis planning. This yields the question of whether one can use a general synthesizability score in *de novo* design first and solve with in-house building blocks afterward to receive the same candidates. For this purpose, we created a joint UMAP projection [32] of all the solved and potentially active candidate molecules from both the in-house and general synthesizability scores trained with Caspyrus10k, making the synthesizability score results comparable as they are trained on the same dataset. Here, molecules generated with these two scores prioritize different chemical sub-spaces, showing that utilizing only a general synthesizability score and running synthesis planning with in-house building blocks afterward is problematic as the generated results can differ (see Fig. 5). Notably, we confirmed the presence of this pattern in high-dimensional fingerprint space by also clustering the combined generated space, resulting in two distinct chemical space clusters for Caspyrus10k that are differentiated by the synthesizability score used during generation (Supplementary: Fig. C3). In detail, the usage of only a general score produces sparse results in areas prioritized by the in-house score and, while still partially recovering the same key scaffolds, creates different molecules. Between both candidate spaces, only 1,124 unique molecules, solved with in-house building blocks and seen as active by the QSAR model, are shared (based on InChI comparisons). For ChEMBL200k, this pattern is also present, though to a lesser extent (Supplementary: Figs. C4, C5).

Hence, when CASP-based synthesizability scores are used as objectives in *de novo* drug design, it is important to note that these scores assess generated molecules based on characteristics influenced by the underlying route planning settings - in our case, the different building blocks used. As demonstrated here, this can greatly impact the chemical space coverage of *de novo* drug design algorithms.

**Fig. 6:**
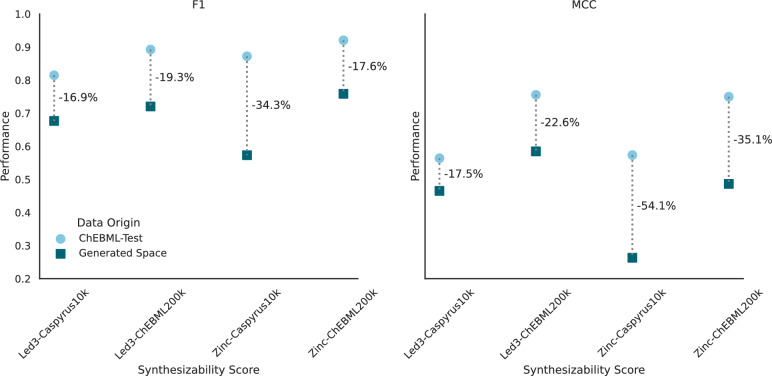
Out-of-distribution predictive performance of synthesizability scores on the explored chemical space. Evaluation of the predictive performance of CASP-based synthesizability scores on *de novo* generated molecules contrasted with the performance on the ChEMBL-Test set (compare Fig. 3). The predictive performance of each score is evaluated by synthesis planning using the building blocks specific to each score’s training

Second, CASP-based synthesizability scores are trained on a specific drug-like chemical space, in our case 200,000 ChEMBL or up to 50,000 Caspyrus molecules, for which synthesis planning is conducted and that is consequently known to the model. However, a specific target chemical space explored by our *de novo* generation might fall outside of this known model scope and produce unreliable predictions. To analyze if this happens in our generation process, we evaluated if our CASP-based scores correctly predict the route planning results for the 100,000 generated molecules and compared the performance to the independent ChEMBL 200k test set (compare Fig. 3). Naturally, we could only compare scores used during the generation with their respective building blocks, meaning that a score trained using synthesis planning results from Zinc building blocks is now also evaluated against Zinc building blocks. Across all models, the performance on generated molecules decreases and performs worse than on the ChEMBL test set, showing a clear domain shift away from the training data (Fig. 6). However, the overall performance for most scores is still acceptable, with around 0.7 F1 and an MCC of around 0.5. For the worst performing Caspyrus10k score based on Zinc building blocks, it is questionable if an MCC of 0.26 is still sufficient to be reliably used.

Overall, these results suggest that synthesizability scores, in-house or general, can be used to generate desired candidates. Nonetheless, it is necessary to exercise caution when using such scores in a *de novo* drug design setting since distinct scores might produce different candidate distributions and as the reliability of the individual scores can differ.

### Experimental candidate and synthesis route evaluation

Next, we experimentally evaluated our methodology regarding the predicted activity and their suggested in-house synthesis routes. For this purpose, we first deployed a virtual screening approach to reduce the candidate set to a manageable size. In detail, we filtered the molecules generated with the in-house Caspyrus10k synthesizability score, requiring that molecules be perceived as active and synthesizable by their respective objective function using a probability filter threshold of 0.8 (32,907 candidates). Next, we reduced the resulting molecules by the requirement that a synthesis route with our in-house building blocks could be found, resulting in 20,055 potential candidate molecules (compare Supplementary: Table C5 for the other scores). It is noteworthy that we relied here on a virtual screening setting rather than directly using the solved candidates from the prior experiments (compare Fig. 4) since this setting reflects a more realistic application of our synthesizability scores in the future, reducing resource-intensive synthesis planning. To decrease the resulting large number of synthesis candidates further, we first analyzed the entire candidate set regarding the Tanimoto similarity for each molecule to the known ligands of MGLL (see Supplementary: Fig. C6). We then applied further filtering in that a found synthesis route cannot be longer than five reaction steps to focus on easy-to-make candidates (4675), required drug-likeness by satisfying the Lipinski rule of 5 [33] (950), and enforced novelty by having a Tanimoto similarity to known ligands of smaller than 0.7 (609). From these 609 candidates, domain experts selected three candidates for experimental validation based on diversity, potential activity (“chemical eye”), and the presence of a short synthesis route (1 or 2 steps). These three candidates were made using the suggested synthesis routes by the synthesis planning algorithm and evaluated in a natural substrate assay for MGLL inhibition.

**Fig. 7:**
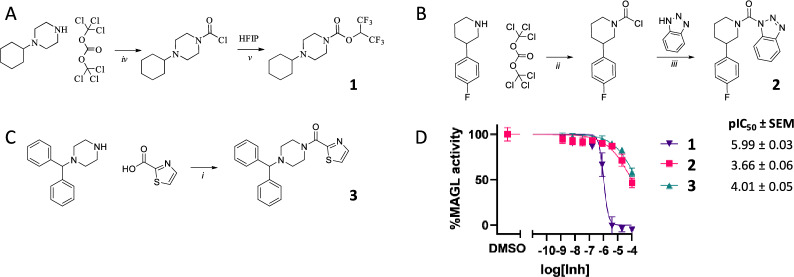
Selected *de novo* generated candidates, synthesis routes based on in-house building blocks, and their experimentally validated activity. **A**–**C** Selected candidates **1**, **2**, **3** for experimental evaluation and their respective in-house synthesis routes. **D**) Residual MGLL enzyme activity after treatment with varying concentrations of inhibitor as measured by natural substrate assay (compare Supplementary: Experimental Evaluation D for details)

The experimental inhibition results of our candidates and their respective in-house synthesis routes are presented in Fig. 7. Compound **1** showed clear activity with an $IC_{50}$ of 1 ${\upmu }$M, and compounds **2** and **3** show slight activity of around 100 ${\upmu }$M $IC_{50}$.

Although all three tested molecules showed some level of inhibitory activity, a stricter boundary of $\le$ 10 ${\upmu }$M, generally used for hit finding, only leaves one candidate that can be classified as active. This somewhat lower potency can be expected, as the selection of molecules was based on conducting at most two synthesis steps, leaving molecules with more expressed side chains and higher potential potency out of the evaluation. Nevertheless, from these experimental results, we can conclude that we can generate in-house synthesizable and active drug candidates that rely on CASP routes using our limited building blocks.

### Critical analysis of *de novo* generated candidates

Given that most *de novo* methods only do an in-silico evaluation of their drug candidates [24], it is vital to critically analyze our experimentally evaluated and active molecules stemming from a *de novo* drug design approach to provide further inside.

**Fig. 8:**
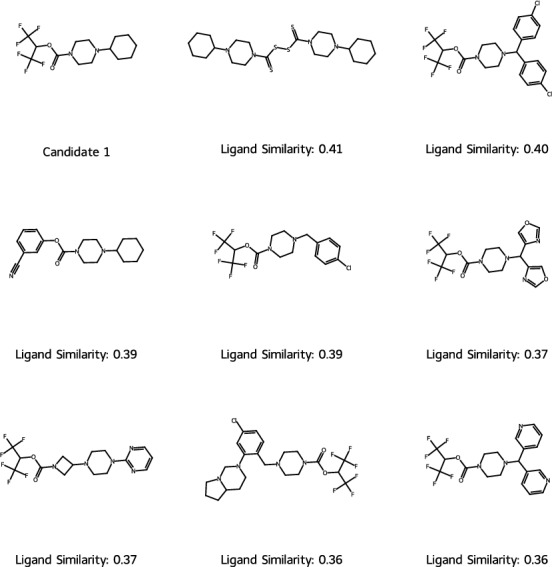
Closest known ligands compared to most active candidate 1. Measured by Tanimoto similarity on Morgan Fingerprints (Radius 3, Size 2048)

For this purpose, we first contrasted our synthesized candidates with known ligands to analyze their novelty. When directly inspecting our selected candidates, even though active and in-house synthesizable, their novelty in key scaffolds is limited. Looking at the closest known ligands, as determined by a Tanimoto similarity threshold, for the respective candidate structures, **2** and **3** are variations of the closest ligand. However, candidate **1**, which was also the most active one in our experiments, deviates more from the closest known ligands in the training dataset and seems to combine distinct motives found in previously explored analogs using the same key scaffold (see Fig. 8, Supplementary: Fig. C7 for candidate **2** & Fig. C8 for candidate **3**), akin to what a medicinal chemist would think of trying in the various Design cycles of a candidate.

**Fig. 9:**
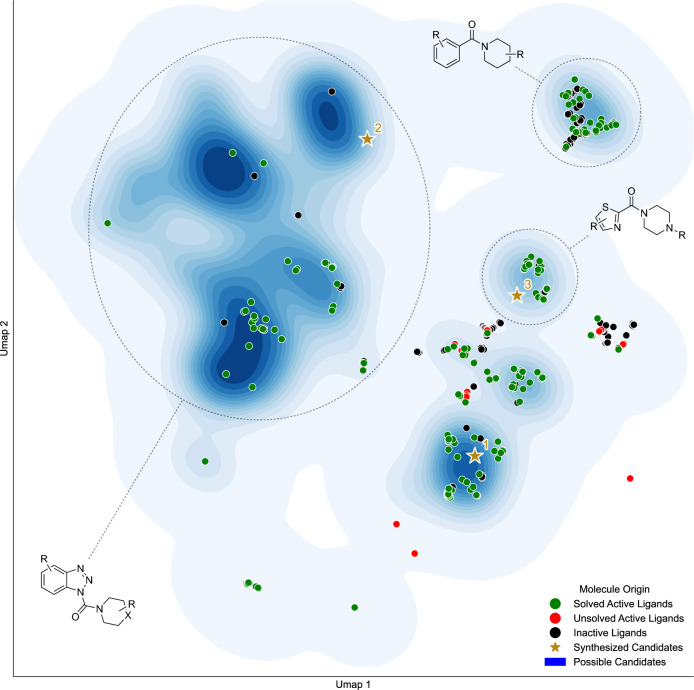
Contrasting the generated drug candidate space with the known MGLL ligand space. UMAP visualization of the solved molecular candidate space of 20,055 molecules generated with in-house synthesizability score (“Caspyrus10k”) and target QSAR model as training objectives and known MGLL ligands. Known MGLL ligands are marked as either inactive (“black circle”) or active. Active ligands are differentiated between synthesizable using in-house building blocks (“green circle”) and those that are not (“red circle”). Experimentally tested candidates are denoted with a star. Exemplary scaffolds are highlighted based on the respective cluster most-frequent Murcko scaffolds. UMAP is calculated using Morgan Fingerprints (Radius 3, Size 2048)

In the second step, we compared our solved candidate space to the known ligands to understand what constitutes our generated space and how our objective functions influence the generation of potential candidates and the presence of key scaffolds. For this purpose, we created a joint UMAP projection of all the solved generated candidate molecules, our three synthesized candidates, and all known ligands for the target. For the known ligands, we annotated which molecules are active or inactive in terms of our QSAR model (compare methods 4.3) and for which of the active ligands a synthesis route could be found with our in-house building blocks. When analyzing the joint UMAP projection of the generated candidate molecules and known ligands (see Fig. 9), candidate molecules are generated in areas where active ligands that are synthesizable with our in-house building blocks are present. From this, we can conclude that the QSAR model works as intended, which is supported by the direct rediscovery of 145 known active ligands in our candidate space (based on InChI comparisons) that the QSAR model also classified as active and, in comparison, the rediscovery of 0 inactive ligands. This, however, also explains the usage of key scaffolds in our generated candidates, as the QSAR model operates on the structures of known ligands for MGLL and does not generalize well beyond that. Inactive known ligands, in comparison, tend to be in areas of low candidate density. They can, however, also be close to active ligands with higher density, especially when analogs to known actives are tested.

We can conclude further that the applied in-house synthesizability score works as intended as a generation objective, as unsolvable active ligands are outside areas with high candidate density. Intriguingly, the model generates two major clusters of molecules with little to no known molecules tested for MGLL. These areas could hold more ’creative’ ligands, which was also illustrated by their lengthier synthetic routes. For synthetic reasons, these were outside of the scope of this research.

## Conclusion

In this work, we have introduced an end-to-end and experimentally evaluated in-house *de novo* drug design approach that provides active drug candidates and their in-house synthesis routes by repurposing already available chemicals to reduce costs, lead times and potentially chemical waste in the drug discovery process.

We have demonstrated that synthesis planning can be successfully conducted by using only a small set of roughly 6000 in-house available building blocks, making it unnecessary to have a commercially vendor-sized stock of building blocks available. With this, we demonstrated the possibility of conducting potential synthesis in-house while repurposing already available resources. Compared to utilizing general vendor building blocks, this in-house approach yields only a –12% decrease in synthesis planning success rate when accepting the resulting, on average, two reactions longer synthesis routes. Next, we leveraged our in-house synthesis planning approach to create an in-house machine learning synthesizability score to predict if a molecule is synthesizable with our in-house building blocks. We further showed that it is possible to train such a score on a small, selected subset of molecules, allowing the recreation of our score within a day in case of changes in our available building blocks, reactions, or the general adaptation to a new laboratory environment by the broader research community. Finally, we showed the successful application of this score in *de novo* drug design by generating molecules that are both active against our selected MGLL target and in-house synthesizable. We further demonstrated that combining synthesis planning and *de novo* drug design is viable and valuable in a small laboratory setting by providing a large set of in-house accessible candidate molecules to our chemists, showing that including such a synthesizability score increased the number of in-house synthetically accessible molecules manifold. Out of this candidate pool, we validated three selected candidates not only in silico but experimentally, finding an active molecule with new disconnection ideas for our target and additionally verifying that the algorithmically proposed in-house synthesis routes are feasible in our laboratory setting.

Even though the proof-of-concept for in-house synthesizability of generated structures is the main focus of this study, a primary limitation relates to the novelty of the generated structures. Generally, we see in our candidates one of the current problems in *de novo* drug design, where key scaffolds for the target are re-used, and the sidechains are algorithmically altered (e.g., [3]). In our work, we do not explore potentially more active candidates with more complex side chains and, consequently, longer synthesis routes, as we find novel ideas for a possible MGLL inhibitor, even when looking only at fairly undecorated molecules. Still, the re-usage of key scaffolds is also present in our work. Even though we do not enforce or fix any scaffolds for the target, our trained molecular generator re-discovers active and in-house synthesizable molecules with known scaffolds on its own.

A natural future improvement is to replace the target QSAR model, potentially limiting the diversity of generated key scaffolds, with other methods for assessing protein-ligand activity like a shape-based pharmacophore [34] or docking [35, 36]. Since both synthesis planning and synthesizability scores are active research fields, improving the synthesis planning performance with more complex neural networks capturing that capture the reaction logic [15, 16] or better approximation models for synthesizability [21, 22] that combine more synthesis route criteria beyond binary CASP-synthesizability [14]. Along the same lines, optimizing the right in-house building blocks to open synthetically accessible chemical spaces might be of further interest. Here, the presence of the right mix of small laboratory building blocks could allow the synthesis of a broader chemical space with as few as possible reactions. Beyond focusing only on in-house synthesizability, merging in-house with cheap and easy-to-acquire vendor building blocks could be of practical interest to maximize cost-efficient synthesis.

Finally, our in-house synthesizability score is regularly used in our university setting for *de novo* drug design and virtual screening to streamline the overall drug discovery process. Its internal usage and the application of similar scores in other institutions will hopefully facilitate a change for a more efficient and sustainable drug discovery process and a further combination of contemporary artificial intelligence methods with real-world laboratory experimentation going forward. For this purpose, we provide all relevant code that relies solely on open-source software and all data to reproduce the results presented in this work, allowing easy and cost-free creation of other in-house synthesizability scores.

## Methods

### Synthesis planning

For all synthesis planning in this study, we used the publicly available open-source AiZynthfinder [11, 37] synthesis planning framework. Specifically, we relied on the AiZynthfinder-provided NeuralSym reaction network [38] that is trained on publicly available USPTO reactions [39] and Monte-Carlo Tree Search [7] as the respective search algorithm. The search settings were limited to a search time of 900 s per molecule, 1000 search iterations, and a synthesis route depth of 8. Further, we added 50 possible reactions to the tree search per reaction model call (compare Supplementary: Table A2 for details). The building blocks used, i.e., search targets in the tree search, were 17,422,831 Zinc building blocks provided by AiZynthinder [11], used for the general evaluation of synthesizability, and 5,955 building blocks provided by the Leiden University Early Drug Discovery & Development department [40], used for in-house synthesizability.

We utilized two datasets to evaluate synthesizability using the respective building blocks: First, we created a representative subset of the synthesizable drug-like molecules space that allows fast evaluation and retraining of potential synthesis scores named Caspyrus. The creation process mimicked our work evaluating different model architectures in synthesis planning with 10,000 molecules [16]. We selected the high-quality Papyrus dataset [26] of 1,238,835 molecules and cleaned them with the Guacamol cleaning strategy [23] to ensure drug-like molecules. We further removed known building blocks stemming from Zinc [11], Enamine [41], MolPort [42] and eMolecules [43]. We then clustered the remaining molecules using Butina clustering [27] with a cut-off of 0.6 using Morgan fingerprints [44] (radius of 2, fingerprint size of 1024), which resulted in 137,963 cluster centroids. From these centroids, we removed 19 centroids that are directly in clinical study phases 1–3 [45] as we wanted to prevent later molecular generation towards intellectual property spaces. Finally, we took centroids of the *n* largest clusters to create the different Caspyrus versions (see Table 1).

**Table 1:** Different Caspyrus versions

Name	Centroids	Molecules
Caspyrus1k	1000	82,352
Caspyrus10k [16]	10,000	280,956
Caspyrus20k	20,000	371,231
Caspyrus50k	50,000	491,422

Overview of the selected cluster centroids per Caspyrus dataset and their overall represented molecules

Second, we sampled 200,000 molecules from ChEBML, following the evaluation framework of RaScore [20], and cleaned them with the same Guacamol cleaning strategy. Compared to the clustered Caspyrus dataset, this dataset is more likely to contain noisy data, duplicates, and potential building blocks.

We measured the number of molecules for which at least one complete synthesis route with the respective building block sets could be found on both evaluation datasets. Furthermore, we used the shortest found route of all found synthesis routes to evaluate the minimum route length.

### Synthesizability scores

We leveraged the results of the synthesis planning to train our general and in-house synthesizability scores. To approximate synthesis planning, we used XGBoost [29] as a binary classifier to learn the relationship between the selected molecules and their synthesis planning result (synthesis route found/not found). We selected the rather “simplistic” XGBoost, following the well-working RaScore [20], as we were more interested in the general applicability of our approach and because more complex Graph Neural Network architectures showed only slight performance improvements [21, 22]. The input into all XGBoost models were Morgan fingerprints (radius of 3, size of 2048) using additional selected chemical properties following DrugEx [19].

All classifiers were trained and evaluated with the following scheme: Initially, we split away 10% of the respective data as a test set following the process of RaScore [20], where we used the ability to find a synthesis route with Led3 building blocks as a stratifying criterion. On the remaining 90% of the data, the training dataset, we conducted a 5-fold cross-validation to evaluate different hyperparameter settings. Our hyperparameter optimization scheme consists of 1000 rounds of Bayesian Optimization for every classifier using Bayesian Optimization and Hyperband [46] - in total, multiple days of runtime per classifier. Here, the selected hyperparameters were the learning rate (0.05$-$0.4), maximum depth of a tree (1–50), minimum loss reduction required for further partition of a tree (0–10), and number of trees (5–250). The final score is then trained on the entire training dataset using the best hyperparameters.

The final performance of each score is evaluated on two datasets: First, the respective 10% test data for each dataset not used during training. Second, we sampled an additional 200,000 cleaned molecules from ChEMBL [28] and conducted synthesis planning to create a new test to measure the generalizability of the trained scores on a large chemical space (compare Supplementary: Table B3 for optimal hyperparameter settings and results). Noteworthy, we ensured that the molecules from this ChEMBL test set are neither in the Caspyrus nor the Chembl200k datasets used to train our CASP-based synthesizability scores.

### De novo molecular generation

The trained CASP-based synthesizability scores were evaluated in a *de novo* drug design setting, where the goal was to generate active and in-house synthesizable molecules for our selected MGLL protein target [25], evaluated by in silico synthesis planning and experimental evaluation.

For this purpose, we used our molecular generator DrugEx [19] alongside a set of desirable generation objectives, in our case, a trained target QSAR model and multiple different synthesizability scores. We selected DrugEx v3 as the molecular generator for two reasons: First, DrugEx is currently the only Reinforcement Learning (RL) approach that uses a reward based on the Pareto front instead of a single or a scalarized objective [47], which allows the model to more accurately learn the trade-offs between different objectives and produce more diverse solutions. This is especially important in our setting as the predicted biological activity by the QSAR model and our selected synthesizability scores are non-consumable without losing information about the trade-offs between both objectives, meaning that molecules evaluated to be active are not necessarily also assessed to be synthesizable and vice versa. Second, we hope for the adoption of our approach in the future, as DrugEx is open source, well-maintained with high code quality [48] and allowed for all the data and methods used to create this work to be publicly available. Given that the DrugEx framework offers several generative model architectures, we decided to use the latest graph-based transformer model operating on fragments in this work [19], where the goal is to learn the generation of novel and valid molecules from a predetermined chemical space given a set of starting fragments–substructures smaller than known key scaffolds. The version 3.4.0.dev1 of the DrugEx software was used throughout this work.

In our case, the training process of DrugEx consisted of three steps:

1. A pretrained model was obtained, that captures the general drug-like chemical space by learning the mapping between fragments and their respective molecules. Here, we used a pre-trained model based on Papyrus 05.5 [26] that was trained by applying BRICS fragmentation [49] on the molecules in Papyrus to achieve the aforementioned goal.
2. A fine-tuned DrugEx model was created by conducting transfer learning on the pre-trained model with the chemical space related to MGLL. For this purpose, we extracted 700 structures related to MGLL from Papyrus 05.5 [26] using the MGLL Uniprot ID Q99685 (Supporting information: Q99685.tsv) and utilized them to fine-tune the pre-trained model. These 700 ligands in the fine-tuning set were also fragmented with the BRICS method following the same protocol as the pre-trained model (1). Out of the resulting data set of fragment-molecule pairs, 10% were used for validation and implementation of the early stopping strategy. The training process ran for 200 epochs with a batch size of 512 until no improvement in loss could be observed after 50 epochs (compare Supplementary: Fig. C10).
3. In the final step, we used RL to steer our model towards generating active and synthesizable molecules by repeatedly generating a set of molecules, evaluating the generated molecules with our objectives, and retraining the model based on the Pareto-front of both active and synthesizable molecules. Here, the general pre-trained model (1) was used as the actively trained network ($G_\vartheta$) and the fine-tuned model (2) as the fixed network ($G_\varphi$) in the DrugEx RL exploration strategy [19]. To train the model, the same set of training and validation fragment-molecule pairs was used as in the fine-tuning step (2). Given that we wanted to evaluate the effect of different synthesizability scores, we trained multiple models that each combined a different synthesizability score with our QSAR model (see Table 2). Further, several values for the exploration parameter epsilon were explored that controlled the fraction of data originating from the fixed fine-tuned ligand space model during training (compare Supplementary: Fig. C11). For all objectives, modifier settings were set according to values recommended in the literature or based on a suitable classification threshold to support smooth model training (compare Supplementary: Table C7). For each trained model, the training was set to continue for at most 500 epochs, with early stopping being triggered once the overall desirability on the validation set stopped improving. Based on the epsilon trade-off data obtained (compare Supplementary: Fig. C11), the final set of 100,000 compounds was generated with models with an exploration parameter epsilon of 0.2 as they offered the best trade-off between objective optimization (desirability) and structural diversity. All models built are made available in the public domain as part of the provided data.

**Table 2:** Trained DrugEx models

Synthesizability Score	Training Data	Building Blocks
QSAR Only	–	–
SAScore	–	–
Led3Caspyrus10k	Caspyrus10k	In-house
Led3ChEMBL200k	ChEMBL200k	In-house
ZincCasyprus10k	Casyprus10k	General
ZincChEMBL200k	ChEMBL200k	General

Models are trained using a combination of the QSAR model alongside a synthesizability score, relying in the case of CASP-based synthesizability scores on a unique set of training data and building blocks

**Fig. 10:**
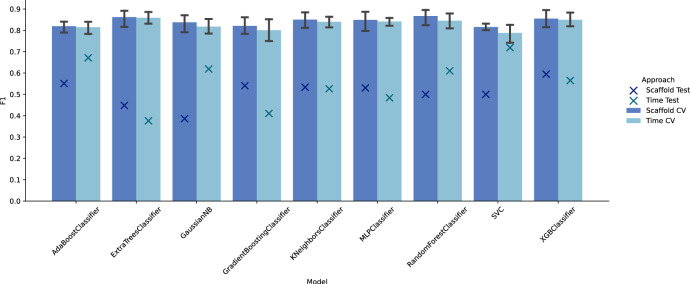
Performance of the QSAR model evaluated on known MGLL ligands. Performance is measured using 5-fold cross-validation on the training data (“CV”) and an independent test dataset (“Test”) while employing both scaffold- and time-splits

The QSAR model used for the MGLL [25] activity objective was trained by using the QSPRPred library [50], which directly interfaces with DrugEx to facilitate QSAR model scoring. The same set of 700 MGLL ligands from Papyrus, as described in the fine-tuning step (2), was used to obtain bioactivity data for this model. For model evaluation and selection, we divided the ligands into training and test sets using both a scaffold split (80% training, 20% test) and a time split (pre-2018 training, since 2018 test), comparing the results obtained from different models under both evaluation strategies. Here, we opted for a classification task instead of a regression task for the QSAR modeling as, from our experience, classification works better in DrugEx during RL optimization. The labels to distinguish active and inactive molecules were taken from the pChEMBL values in Papyrus, where molecules with at least 6.5 pChEMBL were treated as active. For both scaffold- and time-splits, we applied hyperparameter optimization using grid-search with a 5-fold cross-validation on the training data (compare Supplementary: Table C8) to find the optimal hyperparameters and selected the best model algorithm based on the overall test-set performance across both evaluation strategies. Out of the nine evaluated models via QSPRPred (Random Forrest, Extra Tree Classifier, XGBoost, Multi-Layer Perceptron, Gradient Boosting Classifier, AdaBoost, k-nearest neighbors, Support Vector Classification, and Gaussian Naïve Bayes) [29, 51], we picked XGBoost for our QSAR model as it performed consistently well across both the scaffold and time split benchmarks (see Fig. 10) and provided fast inference speeds required for our RL training. Due to data scarcity, we retrained the selected XGBoost classifier afterward with all known bioactivity data for our target. The optimal hyperparameters for this final model were chosen from the prior scaffold-split optimization workflow, as the resulting model showed the best performance both during cross-validation and on the external test set.

To investigate the effect of our synthesizability scores on generated molecules, we used different synthesizability scores as a second objective alongside the QSAR model (see Table 2). In our baseline setting, we only used the QSAR model without any synthesizability score (“QSAR Only”) or combined SAScore [18] with the QSAR model (“SAScore”). We picked SAScore as a heuristic synthesizability baseline as it is a widely adopted measure to evaluate molecules (e.g., [23]) and differs substantially from our CASP-based synthesizability scores as it measures the topological complexity of a molecule instead of approximating the ability to find a synthesis route using synthesis planning. As SAScore does not provide a probability for synthetic complexity, we transformed the scores using a smoothed-clipped score function (compare Supplementary: Table C7). For our non-baseline setting, we selected four different CASP-based synthesizability scores alongside our QSAR model, where two measured the in-house synthesizability and the other two measured general synthesizability. For our in-house synthesizability scores, we used models trained on the Caspyrus10k and ChEMBL200k datasets using in-house building blocks. The rationale behind this selection was two-fold: First, we wanted to know how much data is required to train a synthesizability score. Second, a synthesizability score based on 10,000 molecules is easily retrainable in case of available building blocks or reaction changes, as the computational requirements of running synthesis planning differ substantially between 10,000 and 200,000 molecules. For the general synthesizability scores, we selected models based on the same Caspyrus10k and ChEMBL200k datasets, as this allowed a direct comparison on the same training dataset between our sparse locally available in-house building blocks and generally available building blocks. Noteworthy, the ChEMBL200k score mimics the RaScore [20], as it is trained with the same amount of data and comparable building blocks.

To evaluate different combinations of the QSAR model and synthesizability score, we generated 100,000 molecules for each uniquely trained DrugEx model. We evaluated the synthesizability of our generated molecules by conducting synthesis planning using in-house and general building blocks on the generated molecules with the same settings as in the prior synthesis planning step. Given that we can sample indefinitely from our trained models, we sampled 100,000 molecules for each trained model, assuming that a denser population of candidates generated along the Pareto front should increase our hit probabilities (e.g., [47]) and provide us with enough examples to evaluate each score profusely.

## Supplementary Information

**Supplementary materials 1.**

## Data Availability

All source code, models and relevant data of this work can be found at https://github.com/AlanHassen/led3score.
