# Supplementary material for: Generate what you can make: achieving in-house synthesizability with readily available resources in de novo drug design
Source: J Cheminform. 2025 Mar 28;17:41. doi: 10.1186/s13321-024-00910-4 (PMC11954305; doi:10.1186/s13321-024-00910-4)
Supplement: Supplementary file 1 — Supplementary materials 1. [file 13321_2024_910_MOESM1_ESM.pdf]

Appendix A In-house synthesis planning

Table A1 Synthesis Planning Performance. Evaluation using 5,955 Leiden University in-house (“Led3”) or 17.4 million general building blocks (“Zinc”). Percentage of molecules where a complete synthesis route to either building blocks can be found using synthesis planning on different subsets of a Butina-clustered subset from Papyrus [26] (“Caspyrus”) or a sample of 200,000 ChEMBL molecules.

	Dataset				
Building Blocks	Caspyrus1k	Caspyrus10k	Caspyrus20k	Caspyrus50k	CHEMBL200k
Led3	52.4%	58.1%	59.3%	59.8%	55.0%
Zinc	69.8%	72.0%	72.1%	71.6%	66.7%

Table A2 Synthesis Planning search settings. AiZynthFinder [11] search settings used throughout this work.

Parameter	Value
Search Algorithm	Mcts
C	1.4
cutoff_cumulative	0.995
cutoff_number	50
Max_transforms	9
Iteration_limit	1000
Time_limit	900
Use_prior	True
Return_first	True
Exclude_target_from_stock	True
Prune_cycles_in_search	True

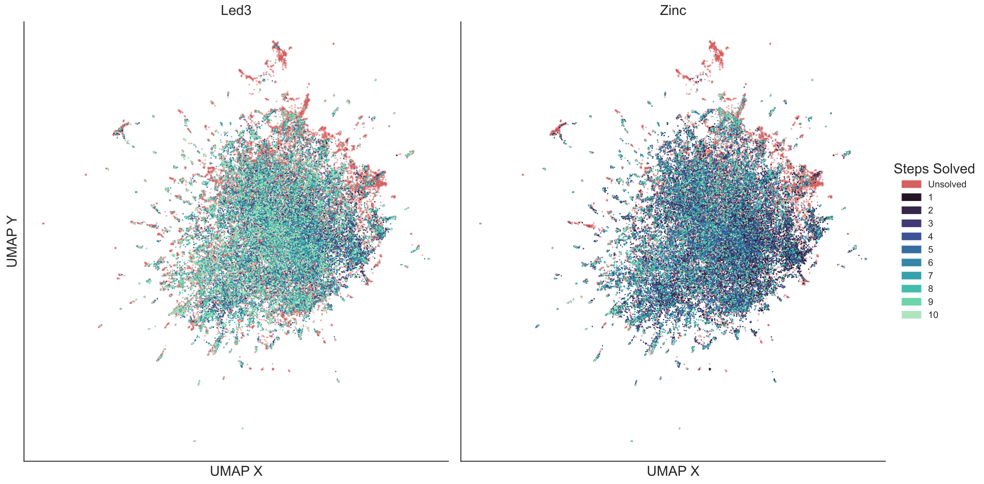

Fig. A1 Comparing route lengths for Led3 and Zinc building blocks on the Caspyrus50k dataset. The figure shows a UMAP visualization of solved and unsolved molecules, with colors representing the respective route lengths for solved molecules. UMAP is calculated using Morgan Fingerprints (Radius 3, Size 2048)

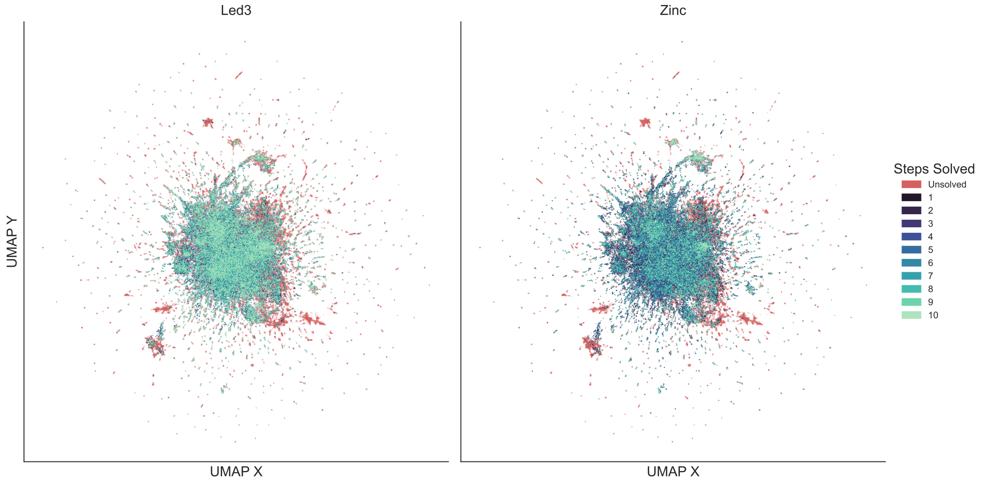

Fig. A2 Comparing route lengths for Led3 and Zinc building blocks on the ChEMBL200k dataset. The figure shows a UMAP visualization of solved and unsolved molecules, with colors representing the respective route lengths for solved molecules. UMAP is calculated using Morgan Fingerprints (Radius 3, Size 2048)

Appendix B In-house synthesizability score

Table B3 Hyperparameter search results for synthesizability scores. Optimal hyperparameters (Learning Rate, Max Tree Depth, Minimum Loss, Numbers of Trees) found for the used XGBoost models and their performance measured by F1 and MCC scores on independent test sets of the respective training datasets ("IND-Test") and 200,000 newly sampled and, to all models, unknown ChEMBL molecules ("ChEMBL-Test").

		Hyperparameter				IND-Test		ChEMBL-Test	
Building Blocks	Dataset	LR	Max. Tree Depth	Min. Loss	Number of Trees	F1	MCC	F1	MCC
Led3	Caspyrus1k	0.272	1	4.950	205	0.721	0.379	0.763	0.409
	Caspyrus10k	0.269	18	0.009	244	0.823	0.563	0.815	0.564
	Caspyrus20k	0.196	17	0.039	244	0.843	0.604	0.828	0.601
	Caspyrus50k	0.200	17	0.016	223	0.867	0.661	0.846	0.645
	ChEMBL200k	0.228	18	0.093	241	0.893	0.758	0.892	0.756
Zinc	Caspyrus1k	0.156	34	0.220	247	0.857	0.268	0.846	0.465
	Caspyrus10k	0.112	14	0.061	221	0.889	0.540	0.872	0.573
	Caspyrus20k	0.097	14	0.266	221	0.892	0.571	0.880	0.604
	Caspyrus50k	0.141	16	0.056	243	0.910	0.657	0.890	0.648
	ChEMBL200k	0.167	18	0.090	245	0.924	0.760	0.920	0.750

Appendix C In-house de novo drug design

Table C4 Generated synthesizable and potentially active molecules using in-house synthesizability scores. Evaluation of 100,000 molecules generated per selected QSAR model and CASP-based synthesizability score combination. "Solved" denotes the successful identification of a synthesis route for a particular molecule with the respective building blocks (in-house Led3 and Zinc), while "Active" is measured by the QSAR model with a probability threshold of greater than 0.8.

Synthesizability Score	Building Blocks	Solved	Solved & Active
QSAR Only	Led3	1,468	762
QSAR Only	Zinc	1,635	883
SAScore	Led3	58,052	2,447
SAScore	Zinc	62,452	2,851
Led3-Caspyrus10k	Led3	35,697	25,044
Led3-Caspyrus10k	Zinc	42,564	30,071
Zinc-Caspyrus10k	Led3	35,102	24,912
Zinc-Caspyrus10k	Zinc	35,084	24,575
Led3-ChEBML200k	Led3	30,650	22,202
Led3-ChEBML200k	Zinc	30,765	21,732
Zinc-ChEBML200k	Led3	43,655	24,109
Zinc-ChEBML200k	Zinc	48,078	26,554

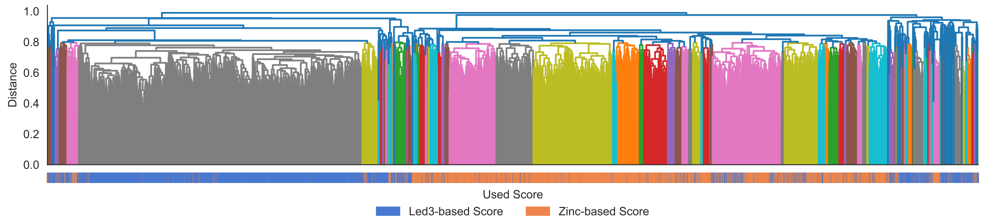

Fig. C3 Clustering the shared generated chemical space of in-house and general synthesizability scores using Caspyrus10k. Hierarchical clustering (complete-linkage) is performed on 10,000 randomly sampled molecules from the solved and potentially active molecular space derived by combining molecules generated using in-house and general synthesizability scores trained on the same dataset ("Caspyrus10k"). In both instances, in-house building blocks are used for synthesis planning to evaluate solvability. Distances are calculated using the Jaccard distance based on Morgan Fingerprints (Radius 3, Size 2048).

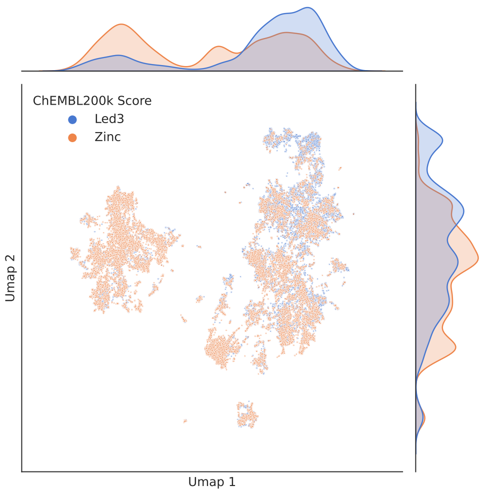

Fig. C4 Contrasting the shared generated chemical space of in-house and general synthesizability scores using ChEMBL200k. UMAP visualization of the solved and potentially active molecular space derived from combining the molecules generated from both in-house and general synthesizability scores trained on the same dataset ("ChEMBL200k"). In both instances, in-house building blocks are used for synthesis planning to evaluate solvability. UMAP is calculated using Morgan Fingerprints (Radius 3, Size 2048).

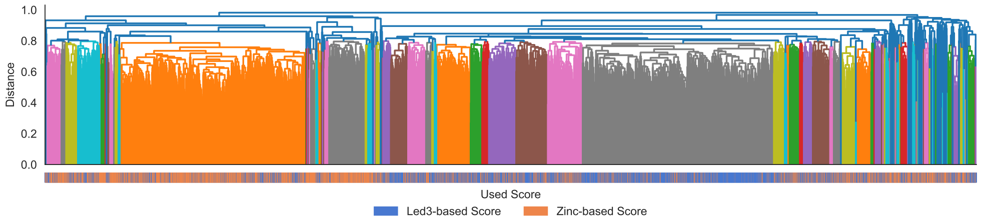

Fig. C5 Clustering the shared generated chemical space of in-house and general synthesizability scores using ChEMBL200k. Hierarchical clustering (complete-linkage) is performed on 10,000 randomly sampled molecules from the solved and potentially active molecular space derived by combining molecules generated using in-house and general synthesizability scores trained on the same dataset ("ChEMBL200k"). In both instances, in-house building blocks are used for synthesis planning to evaluate solvability. Distances are calculated using the Jaccard distance based on Morgan Fingerprints (Radius 3, Size 2048).

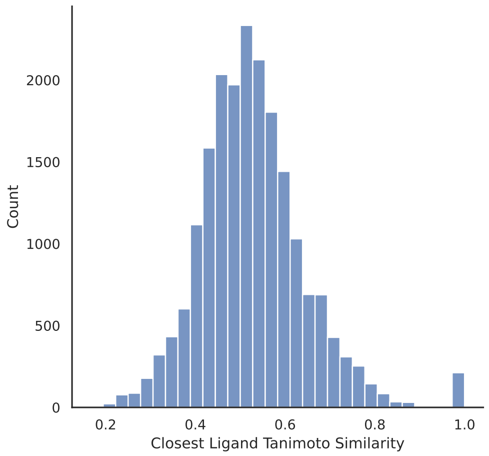

Fig. C6 Tanimoto Similarity to the closest known ligands. Evaluated on the solved virtual screening results using the in-house Caspyrus10k model, determined using Morgan Fingerprints (Radius 3, Size 2048).

Table C5 Virtual Screening results with post-generation filtering. Virtual Screening outcomes for the 100,000 generated molecules from each trained DrugEx model, employing training objectives as post-generation filters. The 100,000 generated molecules are filtered by the QSAR model, the respective synthesizability score, and both combined (desired molecules). These desired molecules are evaluated with synthesis planning using both Led3 and Zinc building blocks. Filter thresholds are set at > 0.8 for both the QSAR and synthesizability models and ≤ 4.5 for the SAScore.

	QSAR Model Filter			Desired Solved	
RL Training Objectives	QSAR Only	SAScore	Both (Desired)	Led3 BB	Zinc BB
QSAR Only	76,828	-	-	762	883
SAScore	7,689	95,331	6,089	2,420	2,808
Led3-Caspyrus10k	67,407	48,093	32,907	20,055	23,054
Led3-ChEMBL200k	64,913	26,229	18,307	14,077	13,597
Zinc-Caspyrus10k	66,628	66,664	48,338	22,419	22,058
Zinc-ChEMBL200k	51,310	54,668	27,775	19,780	21,440

Table C6 Out-of-distribution predictive performance of synthesizability scores on the explored chemical space. Evaluation of the predictive performance of CASP-based synthesizability scores on de novo generated molecules. The predictive performance of each score is evaluated by synthesis planning using the building blocks specific to each score's training.

Synthesizability Score	Accuracy	Precision	Recall	F1	MCC
Led3-Caspyrus10k	0.696	0.545	0.892	0.677	0.465
Led3-ChEMBL200k	0.809	0.653	0.804	0.720	0.585
Zinc-Caspyrus10k	0.496	0.408	0.965	0.573	0.263
Zinc-ChEMBL200k	0.716	0.641	0.929	0.759	0.486

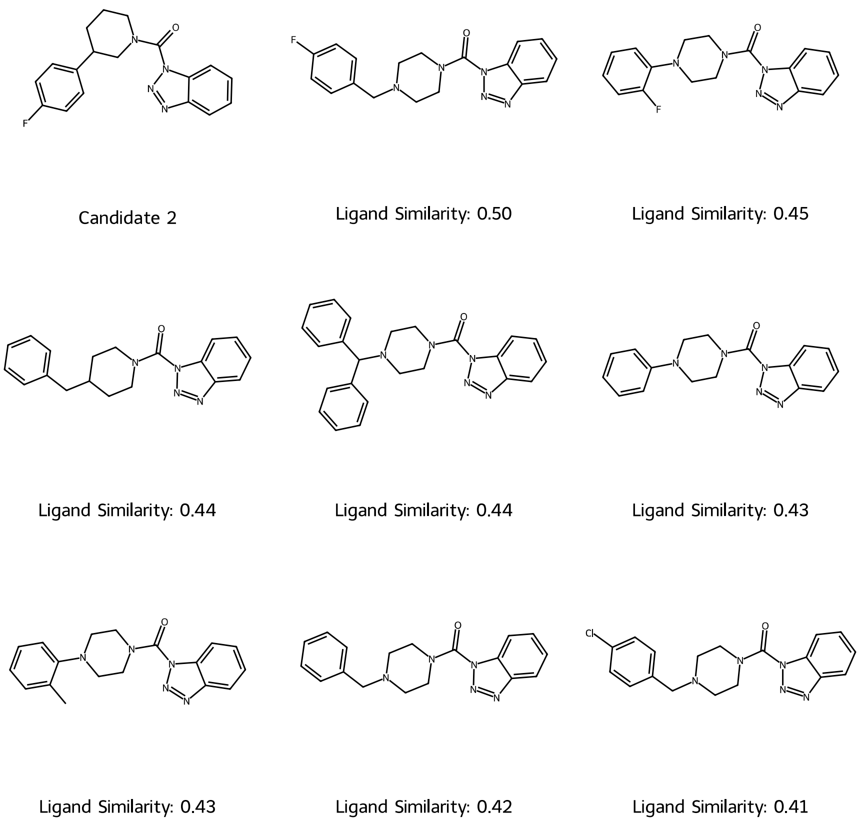

Fig. C7 Closest known ligands compared to most active candidate 2. Measured by Tanimoto similarity on Morgan Fingerprints (Radius 3, Size 2048).

Table C7 DrugEx RL objective modifier functions and class decision thresholds. This table details the parameters of different modifier functions used within DrugEx, including their lower and upper bounds (Lower_x, Upper_x) and class decision thresholds. Detailed descriptions of modifier functions are available in Table S2 [48].

DrugEx Objective	Modifier Function	Lower_x	Upper_x	Class Decision Threshold
QSAR Classifier	ClippedScore	0.2	0.8	0.5
SAScore	SmoothClippedScore	7	4	0.5
Led3-based Score	ClippedScore	0.2	0.8	0.5
Zinc-based Score	ClippedScore	0.2	0.8	0.5

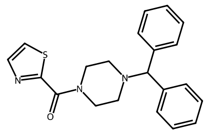

Candidate 3

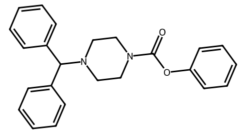

Ligand Similarity: 0.47

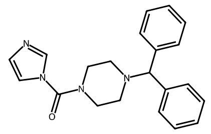

Ligand Similarity: 0.46

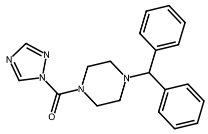

Ligand Similarity: 0.44

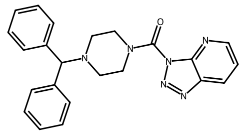

Ligand Similarity: 0.44

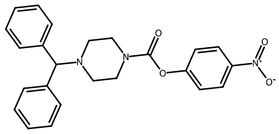

Ligand Similarity: 0.41

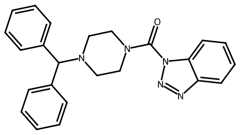

Ligand Similarity: 0.40

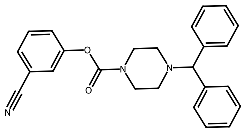

Ligand Similarity: 0.39

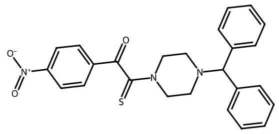

Ligand Similarity: 0.38

Fig. C8 Closest known ligands compared to most active candidate 3. Measured by Tanimoto similarity on Morgan Fingerprints (Radius 3, Size 2048).

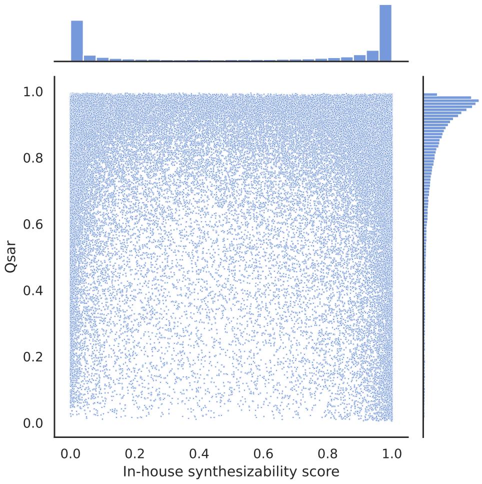

Fig. C9 Pareto Front of the generated molecules using DrugEx. QSAR indicates the perceived activity with respect to our protein target, in-house synthesizability score indicates the synthesizability perceived by our in-house Caspyrus10k synthesizability score.

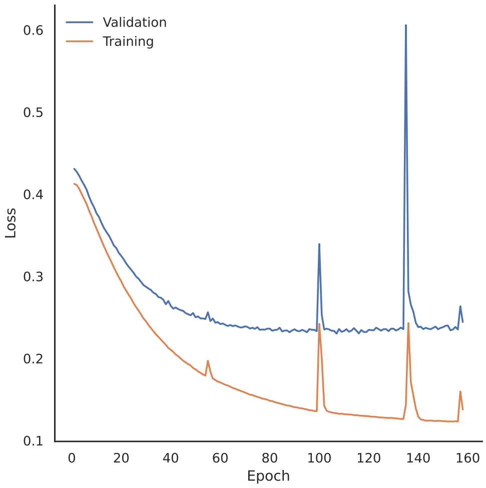

Fig. C10 DrugEx Fine-Tuning Loss. Validation and Training loss during DrugEx fine-tuning of the domain-specific ligand space model.

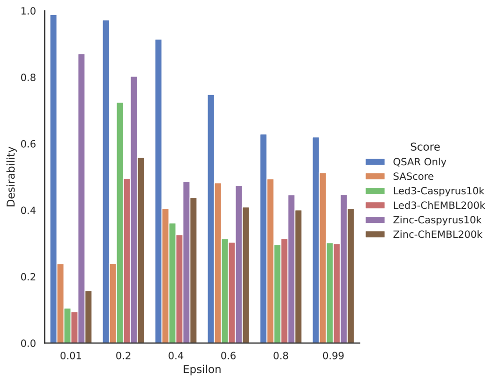

Fig. C11 DrugEx epsilon parameter trade-offs. Maximum Desirability achieved by each model during RL for varying values of the exploration parameter epsilon. The parameter epsilon captures the fraction of data that is sampled during each training iteration from the fixed fine-tuned ligand space model instead of the actively trained model.

Table C8 QSAR model hyperparameter optimization. Overview of tested classifiers, different hyperparameters, possible grid search values and the best-found values.

Algorithm	Hyperparameter	Possible Values	Best
RandomForest	n_estimators	50, 200, 1000	1000
	criterion	gini, entropy, log_loss	log_loss
	min_samples_split	30, 2, 0.1, 0.05	2
	min_samples_leaf	30, 1, 0.1, 0.05	1
	max_features	sqrt, log2	sqrt
	class_weight	balanced, balanced_subsample, None	balanced
	max_samples	0.3, 0.7, 1.0	1.0
	ccp_alpha	0.005, 0.05, 0.1	0.005
ExtraTrees	n_estimators	50, 200, 1000	200
	criterion	gini, entropy, log_loss	log_loss
	min_samples_split	30, 2, 0.1, 0.05	2
	min_samples_leaf	30, 1, 0.1, 0.05	1
	max_features	sqrt, log2	sqrt
	class_weight	balanced, balanced_subsample, None	balanced
	max_samples	0.3, 0.7, 1.0	1.0
	ccp_alpha	0.005, 0.05, 0.1	0.005
XGBoost	learning_rate	0.001, 0.01, 0.1, 0.3, 1.0	0.1
	max_depth	5, 10, 50, 100	50
	n_estimators	50, 200, 1000	50
	colsample_bytree	0.3, 0.7, 1.0	0.3
	colsample_bylevel	0.3, 0.7, 1.0	0.3
	colsample_bynode	0.3, 0.7, 1.0	1.0
	lambda	0.0, 0.5, 1, 2, 5	0
	alpha	0.0, 0.5, 1, 2, 5	0
Multi-Layer Perceptron	hidden_layer_sizes	(50,), (50, 50), (100,), (100, 100), (500,), (500, 500), (500, 100), (100, 500)	(100, 500)
	alpha	0.0001, 0.001, 0.01	0.001
	early_stopping	True, False	False
	max_iter	50, 100, 200, 1000	50
GradientBoosting	n_estimators	50, 200, 1000	200
	min_samples_split	30, 2, 0.1, 0.05	0.1
	min_samples_leaf	30, 1, 0.1, 0.05	1
	max_features	sqrt, log2	sqrt
	ccp_alpha	0.005, 0.05, 0.1	0.005
	loss	log_loss, exponential	exponential
	learning_rate	0.001, 0.01, 0.1, 0.3, 1.0	0.1
	subsample	0.3, 0.7, 1.0	0.7
	n_iter_no_change	1, 5	5
	tol	0.0001, 0.001, 0.01, 0.1	0.0001
AdaBoost	n_estimators	50, 200, 1000	1000
	learning_rate	0.1, 1.0, 2.0, 5.0	0.1
KNN	n_neighbors	1, 3, 5, 10	5
	weights	uniform, distance	distance
	metric	cityblock, manhattan, euclidean, cosine	cityblock
SVC	C	0.5, 1.0, 5.0	5.0
	kernel	linear, poly, rbf, sigmoid	linear
Gaussian Naive Bayes	var_smoothing	1e-9, 1e-6	1e-6

Appendix D Experimental Evaluation

D.1 Biochemistry experimental

D.1.1 Cloning, overexperssion and membrane preparation

Full-length cDNA encoding human MGLL (GenBank ID: BC006230.2; obtained from Source Bioscience) was amplified by PCR and cloned into expression vector pcDNA3.1 in frame with a C-terminal FLAG-tag. All plasmids were isolated from transformed XL10-Gold competent cells (prepared using E. coli transformation buffer set; Zymo Research) using plasmid isolation kits following the supplier's protocol (Qiagen). Constructs were verified by Sanger sequencing (Macrogen).

HEK293T (human embryonic kidney) cells were obtained from ATCC and tested on regular basis for mycoplasma contamination. Cultures were discarded after 2-3 months of use. Cells were cultured at 37 °C under 7% CO_2 in high-glucose DMEM containing phenol red, stable glutamine, 10% (v/v) high iron newborn calf serum (Seradigm), penicillin and streptomycin (200 μg/mL each; Duchefa). Medium was refreshed every 2-3 days and cells were passaged two times a week at 80-90% confluence. One day prior to transfection, HEK293T cells were transferred from confluent 10 cm dishes to 15 cm dishes. Before transfection, medium was refreshed (13 mL). A 3:1 mixture of polyethyleneimine (PEI; 60 μg/dish) and plasmid DNA (20 μg/dish) was prepared in serum-free medium (2 mL) and incubated for 15 min at RT. The mixture was then added dropwise to the cells, after which the cells were grown to confluence in 72 h. Cells were then harvested by suspension in PBS, followed by centrifugation (200 g, 5 min). Cell pellets were flash-frozen in liquid nitrogen and stored at -80 °C.

Cell pellets were thawed on ice and resuspended in lysis buffer A (20 mM HEPES (pH 7.2), 2 mM DTT, 250 mM sucrose, 1 mM $MgCl_2$, and 25 U/ml benzonase). Suspensions were homogenized by polytron (3 × 7 s, 20,000 rpm, SilentCrusher S; Heidolph, Schwabach, Germany), incubated on ice for 30 min, and subsequently centrifuged at

93,000 g for 30 min at 4°C (Ti70 or Ti70.1 rotor; Beckman Coulter, Woerden, The Netherlands). Pellet was resuspended in storage buffer B (20 mM HEPES (pH 7.2), 2 mM DTT)]. Suspension was homogenized by polytron (1 × 10 s, 20,000 rpm). Protein concentrations were determined with Quick Start Bradford reagent (Bio-Rad, Hilversum, The Netherlands) or Qubit fluorometric quantitation (Life Technologies, Breda, The Netherlands). Membranes were diluted with storage buffer B to the desired concentration, aliquoted, frozen in liquid nitrogen, and stored at -80°C.

D.1.2 Biochemical evaluation of MGLL inhibitors

Assays were performed in HEMNB buffer (50 mM HEPES pH 7.4, 1 mM EDTA, 5 mM $MgCl_2$, 100 mM NaCl, 0.5% (w/w) BSA) in black, flat-bottom 96-well plates (Greiner). Inhibitors were added from 40x concentrated stock solution in DMSO. MGLL-overexpressing membrane preparations (0.3 μg per well) were incubated with inhibitor for 20 min at RT in a total volume of 100 μL. Next, 100 μL assay mix containing glycerol kinase (GK), glycerol-3-phosphate oxidase (GPO), horse radish peroxidase (HRP), adenosine triphosphate (ATP), Amplifu™Red and 2-arachidonoylglycerol (2-AG) was added. Fluorescence (λ_{ex} = 535 nm, λ_{em} = 595 nm) was measured at RT in 5 min intervals for 60 min on a Clariostar (BMG Labtech) plate reader. Final assay concentrations: 1.5 ng/μL MGLL-overexpressing membranes, 0.2 U/mL GK, GPO and HRP, 125 μM ATP, 10 μM Amplifu™Red, 25 μM 2-AG, 5% DMSO, 0.5% ACN in a total volume of 200 μL. For IC_{50} determinations, the assay was performed as described above, but with variable inhibitor concentrations. All measurements were performed in N = 2 (individual plates), n = 2 (technical replicates on same plate) or N = 2, n = 4 for controls. Fluorescence values were corrected for the average fluorescence of the negative control (mock-membranes + vehicle). Slopes of the corrected data were determined in the linear interval. The Z'-factor for each assay plate was calculated using the formula $Z' = 1 - 3(\sigma_{pc} + \sigma_{nc})/(\mu_{pc} - \mu_{nc})$ with σ = standard deviation, μ = mean, pc = positive control and nc = negative control, and plates with $Z' \geq 0.6$

were accepted for further analysis. For IC_{50} determination, slopes were normalized to the positive control and analysed in a non-linear dose-response analysis with variable slope (GraphPad Prism 9.0).

Appendix E Chemistry Experimental

E.1 General chemistry

All used glassware was oven dried. Reagents were either acquired from Sigma-Aldrich, Acros and Merck and used without further purification unless specified otherwise. Moisture sensitive reactions were performed under a nitrogen atmosphere using anhydrous solvents dried over activated molecular sieves (4 Å). Traces of water were removed from starting materials through co-evaporation with toluene. Thin layer chromatography (TLC) was performed using TLC Silica gel 60 F_{245} on aluminums sheets (Merck). Compounds were visualized using an ultraviolet lamp (λ_{max} = 254 nm), $KMnO_4$ staining (K_2CO_3 (40 g), MnO_4 (6 g), H_2O (600 mL) and 10% NaOH (5 mL)) or ninhydrin staining (ninhydrine (200 mg), AcOH (5 mL) and EtOH (100 mL). The crude compounds were purified by either flash column chromatography using Screening Devices silica gel 60, or automated flash column chromatography using Biotage Isolera One or Four Flash Chromatography Systems and pre-packed cartridges of Screening Devices UltraPure Irregular Silica Gel (40 – 63 μm, 60 Å). LC-MS measurements were performed on a Thermo Finnigan LCQ Advantage Max ion-trap mass spectrometer (ESI+), coupled to a Surveyor HPLC system (Thermo Finnigan) or a Thermo Vanquish Focused $UHPLC^+$ system, equipped with a C18 column and coupled to a Thermo LCQ Fleet ion-trap mass-spectrometer. Both LC-MS systems were equipped with a standard C18 (Gemini, 4.6 mmD $\times$ 50 mmL, 5 μm particle size, Phenomenex) analytical column. Eluents A: H_2O, B: ACN, C: 1% aq. TFA, gradients: 10 – 90% or a 0 – 50% gradient of ACN in water with 0.1% TFA. A Bruker AV-400 Cryomagnet was used to obtain proton(^{1}H)-NMR and carbon(^{13}C)-NMR. Chemical shifts (δ) are

reported are reported in parts per million (ppm) downfield of tetramethylsilane (TMS) or solvent resonance as the internal standard ($CDCl_3$: δ 7.26 for ^{1}H, δ 77.16 for ^{13}C, CD_3OD: δ 3.31 for ^{1}H, δ 49.00 for ^{13}C). Splitting patterns reported in an abbreviated manner (s = singlet, d = doublet, t = triplet, q = quartet, and m = multiplet), coupling constants (J) are quoted in Hertz (Hz). Peak assignments were aided by 2D COSY, HSQC, and HMBC experiments. MestReNova software (version 14.0.1-23559) was used for the analysis of the NMR spectra. PerkinElmer ChemDraw Professional (version 22.2) was used to draw molecular structures presented in this work.

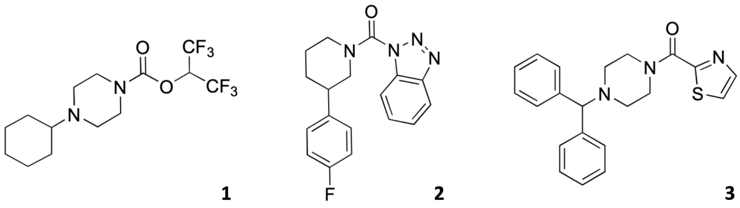

Fig. E12 Experimentally evaluated candidates.

E.1.1 1,1,1,3,3,3-hexafluoropropan-2-yl 4-cyclohexylpiperazine-1-carboxylate (1)

Triphosgene (0.5 eq., 89 mg, 0.30 mmol) and Na_2CO_3 (1 eq., 63 mg, 0.60 mmol) were dissolved in DCM (3.0 mL, 0.1M) under argon and stirred on ice. Subsequently 1-cyclohexylpiperazine (1 eq., 100 mg, 0.60 mmol) in DCM (3 mL) was added and the obtained mixture was stirred at 0 ºC for 1 h. Upon full conversion to the carbamoyl chloride intermediate, the reaction mixture was filtered, rinsed with DCM (10 mL) and concentrated under reduced pressure. Hexafluoroisopropanol (1 eq., 100 mg, 0.60 mmol) and DiPEA (2 eq., 155 mg, 1.20 mmol) were dissolved in DCM (3 mL, 0.2 M) and added drop wisely to the carbamoyl chloride in DCM (3 mL). The obtained solution was stirred under argon atmosphere for 19 h. Upon full conversion of the starting materials, the reaction mixture was quenched with sat. NH_4Cl (2 mL) and

brought to pH 7 using 1M NaOH (1 mL). Subsequently, the neutral solution was extracted with DCM (3x 10 mL), after which the combined organic layers were dried ($MgSO_4$) and concentrated under reduced pressure. After purification with column chromatography eluting with isocratic DCM, the title compound was partially isolated as a white solid (43.0 mg, 0.12 mmol, 20%). ^{1}H NMR (400 MHz, $CDCl_3$) δ 5.75 (p, J = 6.2 Hz, 1H), 3.95 – 3.25 (m, 4H), 2.83 – 2.49 (m, 5H), 2.52 – 2.24 (m, 1H), 2.03 – 1.79 (m, 5H), 1.65 (dd, J = 13.0, 3.6 Hz, 1H), 1.48 – 1.15 (m, 5H), 1.16 – 0.99 (m, 1H). ^{13}C NMR (101 MHz, $CDCl_3$) δ 151.41, 120.80 (t, J = 285.0 Hz), 68.15 (hept, J = 136 Hz), 64.02, 49.07, 48.99, 48.89, 48.66, 48.42, 46.63, 44.93, 44.56, 28.71, 26.20, 25.83.

E.1.2 (1H-benzo[d][1,2,3]triazol-1-yl) (3-(4-fluorophenyl)piperidin-1-yl)methanone (2)

3-(4-Fluorophenyl)piperidine (1 eq., 100 mg, 0.56 mmol) and Na_2CO_3 (1 eq., 46.3 mg, 0.56 mmol) were dissolved in DCM (5.6 mL, 0.1) under argon and stirred on ice. Subsequently, triphosgene (0.5 eq., 82.8 mg, 0.28 mmol) was added and the obtained mixture was stirred at 0 ºC for 1 h. Upon full conversion to the carbamoyl chloride intermediate, the reaction mixture was filtered, rinsed with DCM (10 mL) and concentrated under reduced pressure. To the obtained solid the benzotriazole 1H-benzo[d][1,2,3]triazole (1 eq., 66.5 mg, 0.56 mmol) were dissolved in DCM (2.8 mL, 0.2 M), after which DiPEA (2 eq., 144 mg, 1.12 mmol) was added drop wisely and the obtained solution was stirred under argon atmosphere for 19 h. Upon full conversion of the starting materials, the reaction mixture was quenched with sat. NH_4Cl (2 mL) and brought to pH 7 using 1M NaOH (1 mL). Subsequently, the neutral solution was extracted with DCM (3x 10 mL), after which the combined organic layers were dried ($MgSO_4$) and concentrated under reduced pressure. After purification with column chromatography eluting with isocratic DCM, the title compound was partially isolated as a translucent oil (42.0 mg, 0.13 mmol, 23%). ^{1}H NMR (400 MHz, CD_2Cl_2)

δ 8.04 (dd, J = 42.5, 8.3 Hz, 2H), 7.52 (dt, J = 60.0, 7.6 Hz, 2H), 7.29 – 7.21 (m, 2H), 7.01 (t, J = 8.2 Hz, 2H), 4.62 (d, J = 15.9 Hz, 2H), 3.47 – 2.74 (m, 3H), 2.17 (d, J = 13.7 Hz, 1H), 1.98 – 1.78 (m, 2H). ^{13}C NMR (101 MHz, CD_2Cl_2) δ 161.83 (d, J = 245.0 Hz), 149.47, 145.46, 138.06, 133.28, 129.42, 128.62 (d, J = 7.9 Hz), 125.29, 119.91, 115.56 (d, J = 21.1 Hz), 113.56, 42.12, 31.59, 25.80.

E.1.3 (4-benzhydrylpiperazin-1-yl)(thiazol-2-yl)methanone (3)

The free amine 1-benzhydrylpiperazine (1 eq., 200 mg, 0.79 mmol), PyAOP (2 eq., 597 mg, 1.59 mmol) and the benzoic acid thiazole-2-carboxylic acid (1 eq., 102 mg, 0.79 mmol) were dissolved in DMF (0.4 M), after which DiPEA (4 eq., 0.56 ml, 1.59 mmol) was added dropwisely and the mixture was stirred for 21 h. Upon reaction completion, the solution was dissolved in EtOAc (10 mL) and washed with brine (2 x 10 mL), after which the aqueous layer was extracted with EtOAc (3x 10 mL). The combined organic layers were dried ($MgSO_4$), filtered and concentrated under reduced pressure and the remaining oil was further purified by silica gel column chromatography eluting with a gradient of 0-40% ether in pentane to obtain the title compound as a pale-yellow solid (259 mg, 712 mmol, 90%). ^{1}H NMR (400 MHz, $CDCl_3$) δ 7.77 (d, J = 3.2 Hz, 1H), 7.45 – 7.39 (m, 5H), 7.31 – 7.22 (m, 4H), 7.21 – 7.13 (m, 2H), 4.39 (t, J = 5.0 Hz, 2H), 4.25 (s, 1H), 3.80 (t, J = 5.1 Hz, 2H), 2.48 (dt, J = 11.8, 5.0 Hz, 4H). ^{13}C NMR (101 MHz, $CDCl_3$) δ 165.21, 159.05, 143.03, 142.13, 128.62, 127.89, 127.16, 123.91, 75.96, 52.40, 51.73, 46.55, 43.62.
